# Effects of lysophosphatidic acid on human periodontal ligament stem cells from teeth extracted from dental patients

**DOI:** 10.7555/JBR.32.20170123

**Published:** 2019

**Authors:** Byung Cheol Kim, Jae-In Song, Kyoung-Ha So, Sang-Hwan Hyun

**Affiliations:** 1. Laboratory of Veterinary Embryology and Biotechnology, Veterinary Medical Center and College of Veterinary Medicine, Chungbuk National University, Seowon-gu, Cheongju 28644, Republic of Korea; 2. Institute of Stem Cell & Regenerative Medicine, Chungbuk National University, Seowon-gu, Cheongju 28644, Republic of Korea.

**Keywords:** periodontal ligament stem cell, lysophosphatidic acid, stemness, primary cell culture

## Abstract

Despite their potential applications in future regenerative medicine, periodontal ligament stem cells (PDLSCs) are difficult to obtain in large amounts from patients. Therefore, maintaining stemness while expanding the cell numbers for medical use is the key to transitioning PDLSCs from the bench to the clinic. Lysophosphatidic acid (LPA), which is present in the human body and saliva, is a signaling molecule derived from phospholipids. In this study, we examined the effects of LPA on stemness maintenance in human PDLSCs. Several spindle-shaped and fibroblast-like periodontal ligament stem-like cell lines were established from PDLSC isolation. Among these cell lines, the most morphologically appropriate cell line was characterized. The expression levels of *OCT4*, *NANOG* (a stem cell marker), and *CD90* (a mesenchymal stem cell marker) were high. However, *CD73* (a negative marker of mesenchymal stem cells) expression was not observed. Notably, immunofluorescence analysis identified the expression of *STRO-1*, *CD14*6 (a mesenchymal stem cell marker), and sex determining region Y-box 2 at the protein level. In addition, lipid droplets were stained by Oil red O after the induction of adipogenesis for 21 days, and mineralized nodules were stained by Alizarin Red S after the induction of osteogenesis for 14 days. Alkaline phosphate staining also demonstrated the occurrence of osteogenesis. In summary, we established a human PDLSC line, which could be applied as a cell source for tissue regeneration in dental patients. However, further studies are needed to determine the detailed effects of LPA on PDLSCs.

## Introduction

Third molars or deciduous teeth are often extracted during dental procedures and then discarded. But from the extracted human teeth, stem cells may be obtained[^1^]. Periodontal ligament stem cells (PDLSCs) have potential applications in regenerative medicine as a source for mesenchymal stem cells derived from neural crest cells. Postnatal stem cells can be found in various human tissues, such as the bone marrow, umbilical cord blood, adipose tissue, and muscles[^2^–^3^]. Among these stem cells, PDLSCs is more convenient and less invasive because they can be collected after a simple dental procedure; by contrast, bone marrow-derived stem cells require the patient to undergo a difficult and painful procedure[^4^]. PDLSCs are also associated with fewer immune-related problems because autologous transplantation is possible. Additionally, PDLSCs are free from the ethical problems unlike embryonic stem cells.

In dental research, PDLSCs have attracted attention for their potentials to regenerate and repair damaged dental structures[^3^,^5^]. As shown in previous studies, PDLSCs can differentiate into neurogenic, cardiomyogenic, chondrogenic, and osteogenic lineages, as is needed to form periodontal ligaments, alveolar bone, cementum, peripheral nerves, and blood vessels[^2^,^6^–^8^].

Despite these advantages, PDLSCs can only be obtained in small amounts from a patient. The number of PDLSCs obtained from primary culture is only 1,250 on average[^9^]. However, for periodontal regeneration, at least 4×10^6^ cells are needed[^10^]. Thus, primary cultured PDLSCs must proliferate for at least 12 doublings before clinical use[^11^]. Therefore, maintaining stemness while expanding the cell numbers is essential for transitioning PDLSCs from the bench to the clinic.

Lysophosphatidic acid (LPA), a serum-derived growth factor present in the human body, is a signaling molecule derived from phospholipids. LPA can stimulate cell proliferation, differentiation, migration, and survival[^12^–^15^] and appears to havepro-/anti-inflammatory and pro-/anti-apoptotic properties[^16^]. For example, LPA enhances the survival of human umbilical cord stem cells in ischemic conditions[^17^]. In the dental field, LPA is present in normal human saliva[^18^–^19^] and plasma and may affect the periodontal ligament *in vivo*. Cheng *et al. *have showed that LPA enhances the repair of human dental pulp cells by controlling cell adhesion, migration, and differentiation[^20^–^21^]. Furthermore, LPA rescues human dental pulp cells from ischemia-induced apoptosis by accelerating the repair of injured or damaged tissues[^22^]. LPA also controls regenerative responses of human gingival and periodontal ligament fibroblasts and helps in oral wound healing[^23^]. In addition, a recent study in our laboratory showed positive effects of LPA in porcine female genital cells. A 30 μmol/L LPA treatment during *in vitro* maturation improved the developmental potential of parthenogenic embryos and *in vitro* fertilized embryos in swine.

The Hippo pathway is a newly recognized key regulator of stem cells, organ growth, and tissue repair, and its dysregulation is associated with cancer develop-ment[^24^]. Yes-associated protein (YAP), an effector inhibited by the Hippo pathway, is upregulated in mouse stem and progenitor cells[^25^]. A study of human induced pluripotent stem cells showed that the Hippo pathway blocked reprogramming pluripotency, indicating YAP might regulate pluripotency in pluripotent stem cells[^26^–^27^]. LPA can induce overexpression of YAP, thereby promoting and maintaining naïve pluripotency by suppressing differentiation-inducing effects[^28^].

Based on these findings, we hypothesized that LPA migh tenhance the stemness of PDLSCs. However, LPA also has negative effects, such as promoting tumor growth and bone destruction[^29^]. Therefore, in this study, we examined the effects of LPA on stemness maintenance in human PDLSCs.

## Materials and methods

### Chemicals

All chemicals used in this study were purchased from Sigma-Aldrich Chemical Company (St. Louis, MO, USA), unless otherwise indicated. LPA was dissolved in 0.1% (w/v) BSA in PBS and frozen at −20 ℃ before addition to culture medium.

### Samples and cell culture

This study was approved and supervised by the Institute of Review Board of Chungbuk National University (CBNU-201608-BMBRETC-322-01). Only healthy adults (18 to 30 years old) who provided informed consent for participation were enrolled in this study. Plaque on the teeth was removed with a curette, and the teeth were disinfected with chlorhexidine solution and washed with saline twice. Thenon-decayed third molars were then extracted by a dentist and stored in Dulbecco’s PBS with 1% antimycotic/antibiotic solution (Gibco, Carlsbad, CA, USA). Periodontal ligaments were separated gently from the mid-third of the root, minced into tiny pieces and immersed in a mixture of 1:1 type I collagenase (Worthington Biochem, Freehold, NJ, USA)/dispase II solution. Cells were incubated at 37 ℃ for 60–90 minutes with gentle shaking and isolated to a single-cell suspension using a 70- μm cell strainer (SPL Life Science, Seoul, Republic of Korea). PDLSCs were cultured in Dulbecco’s modified Eagle’s medium (DMEM; Gibco) containing 10% FBS (Gibco) and 1% nonessential amino acids (Gibco), hereafter referred to as growth medium, under humidified conditions of 5% CO_2_ in air at 37 ℃.

### Reverse transcription polymerase chain reaction (RT-PCR)

To determine the mRNA levels of stem cell markers, total mRNA was extracted with TRIzol reagent (Invitrogen Corp., Carlsbad, CA, USA) from cultured PDLSCs at passage 3 according to the manufacturer’s instructions. The total RNA concentration was determined after measuring the absorbance at 260 nm. Complementary DNA (cDNA) was prepared by subjecting 1 μg of total RNA to reverse transcription using Moloney murine leukemia virus (MMLV) reverse transcriptase (Invitrogen Corp.) and random primers (9-mers; Takara Bio, Inc., Otsu, Shiga, Japan). Next, for RT-PCR, the cDNA from hPDLSCs was amplified in a final volume of 20 μL containing 10 pmol of forward and reverse primers, 2 U of Taq polymerase, and 5 pmol of dNTP mixture (iNtRON Biotechnology, SungNam, Republic of Korea). PCR was then performed for 40 cycles under the following conditions: denaturation for 30 seconds at 95 ℃, annealing for 30 seconds at 57 ℃, and extension for 30 seconds at 72 ℃. The reaction products were analyzed on a 1.5% agarose gel prestained with ethidium bromide. The gels were scanned using a Lumino graph Ⅱ (ATTO, Japan), and PCR products were compared with a 100-bp DNA marker (iNtRON Biotechnology).

### Immunofluorescence (IF) analysis

For IF analysis of protein levels in PDLSCs, fixed cells were washed with PBS and permeabilized with 0.2% Triton X-100 for 5 minutes. The cells were blocked with 10% normal goat serum in PBS and incubated with primary antibodies overnight at 4 ℃. The primary antibodies were anti-STRO-1 (MAB4315; Millipore, Temecula, CA; 1:200 dilution), anti-melanoma cell adhesion molecule (MCAM)/CD146 (ABT1488; Millipore; 1:200), anti-sex determining region Y-box 2 (SOX2; AVB5603; Millipore; 1:200). Secondary antibodies were applied and nuclei were stained with Hoechst 33342. The stained cells were examined using a confocal microscope and ZEN 2009 Light Edition software (Carl Zeiss).

### Mesenchymal differentiation and staining

To determine the *in vitro* mesenchymal differentiation potential of PDLSCs, we performed *in vitro* osteogenesis and adipogenesis. After osteogenic and adipogenic differentiation, cells were stained and analyzed for expression of adipogenic and osteogenic marker genes by RT-PCR.

For osteogenesis analysis, 14-day osteogenic induction was conducted using a StemPro Osteogenesis Differentiation kit according to the manufacturer’s protocol. After the 14-day osteogenic induction, cells were fixed with 10% formaldehyde for 10 minutes and stained with 2% Alizarin Red S solution for 20 minutes at room temperature. For alkaline phosphatase staining, cells were fixed with 4% paraformaldehyde for 10 minutes and stained with 50:1 Tris Cl, NBT/BCIP solution (Roche, Basel, Switzerland) for 20 minutes at room temperature. For adipogenesis analysis, 21-day adipogenic induction was conducted using a StemPro Adipogenesis Differentiation Kit according to the manufacturer’s protocol. After 21 days of cell differentiation, adipocyte-like cells were fixed with 4% paraformaldehyde for 10 minutes, incubated with Oil red O working solution for 10 minutes, and observed under a light microscope. After measurement, Oil red O dye was eluted by adding 100% isopropanol and incubating for 10 minutes with gentle shaking. Solutions were transferred to 96-well plates, and the optical density (OD) was measured at 500 nm with 100% isopropanol as a blank using an enzyme-linked immunosorbent assay (ELISA) reader (Epoch, BioTek, VT, USA)[^30^].

### Cell proliferation assays

To evaluate the effects of LPA on PDLSC proliferation, cell proliferation assays were conducted. PDLSCs were seeded at a density of 3×10^3^ cells/well in 96-well plates (Becton Dickinson Labware, Franklin Lakes, NJ, USA) in a humidified atmosphere of 5% CO_2_ at 37 ℃. Cells were incubated with cell culture medium for 24 hours, treated with different concentrations of LPA (0 or 10 μmol/L), and incubated for 1, 3, or 7 days. Cell viability was detected following the addition of Ez-cytox solution (Dogen Bio, Seoul, Republic of Korea). Ez-cytox solution (10 μL) was added to each well of the 96-well plate, and the plates were incubated for 1 hour at 37 ℃. OD was measured at 450 nm using an ELISA reader (Epoch, BioTek, VT, USA) and the number of viable cells was calculated.

### Quantitative RT-PCR (qRT-PCR)

For gene expression analysis, total mRNA was extracted with TRIzol reagent (Invitrogen Corp.) from PDLSCs cultured for 7 days with or without 10 μmol/L LPA according to the manufacturer’s instructions. To determine the conditions for logarithmic-phase PCR amplification of target mRNA, 1-μg aliquots were amplified using differing numbers of cycles. Glyceraldehyde 3-phosphate dehydrogenase (*GAPDH*) was amplified by PCR to rule out the possibility of RNA degradation and control for variations in mRNA concentrations in the RT reaction. Quantitative real-time PCR was performed with SYBR Premix Ex Taq (Takara Bio, Inc.) for 40 cycles using the following cycling parameters: denaturation at 95 ℃ for 15 seconds, annealing at 57 ℃ for 15 seconds, and extension at 72 ℃ for 15 seconds. All oligonucleotide primer sequences are presented in ***Table 1***. The fluorescence intensity was measured at the end of the extension phase of each cycle and the threshold value for the fluorescence intensity of all samples was set manually. The reaction cycle at which the PCR product exceeded this fluorescence intensity threshold was deemed the threshold cycle (Ct) in the exponential phase of PCR amplification. The expression of each target gene was quantified relative to that of the internal control gene (*GAPDH*). The relative quantification was based on a comparison of Ct values at a constant fluorescence intensity. The amount of transcript present was inversely related to the observed Ct. For each two-fold dilution in transcript amount, the Ct was expected to increase by one. The relative expression (R) was calculated using the equation R= 2^−[ΔCt sample−ΔCt control]^. Gene expression values were normalized to those of *GAPDH* and the experiments were repeated at least three times.

**Tab.1:** Primer sequences used for gene expression analysis

Gene Name	Primer sequences	Tm (℃)	Product size (base pairs)	GenBank accession number
**POU5F1**	F: 5’-TGGGGGTTCTATTTGGGAAG-3’	58.4	196	NM_001173531.2
	R: 5’-CTGGTTCGCTTTCTCTTTCG-3’	58.4		
**SOX2**	F: 5’-GACAGTTACGCGCACATGA-3’	58.6	214	NM_003106.3
	R: 5’-GCGAGTAGGACATGCTGTAG-3’	57.9		
**NANOG **	F: 5’-AAAGGCAAACAACCCACTTC-3’	56.4	207	NM_001297698.1
	R: 5’-GGTCTTCACCTGTTTGTAGC-3’	58.4		
**CD90**	F: 5’-CTC TCC TGC TAA CAG TCT TGC-3’	61.3	180	NM_001311160.1
	R: 5’-CCA CAG TGC CAA AGA GCA C-3’	59.5		
**CD34 **	F: 5’-AGCACCAATCTGACCTGAAA-3’	56.4	203	NM_001025109.1
	R: 5’-ATAAGGGTCTTCGCCCAGC-3’	59.5		
**YAP1**	F: 5’-CGGAATATCAATCCCAGCAC-3’	56.4	188	NM_001130145.2
	R: 5’-GAGTGATAGGTGCCACTGTT-3’	57.2		
**PPAR-**γ	F: 5’-CATTATTCTCAGTGGAGACCGC-3’	58.9	191	NM_005037.5
	R: 5’-CACGTGTTCCGTGACAATC-3’	57.0		
**OSTEOCALCIN**	F: 5’-AAGGTGCAGCCTTTGTGT-3’	57.3	188	NM_199173.5
	R: 5’-CCGATAGGCCTCCTGAAAG-3’	57.0		
**GAPDH **	F: 5’--GACTCATGACCACAGTCCAT-3’	56.9	215	NM_001256799.2
	R: 5’-GTCCACCACTGACACGTT-3’	57.2		

## Results

### Morphological analysis

**Fig.1:**
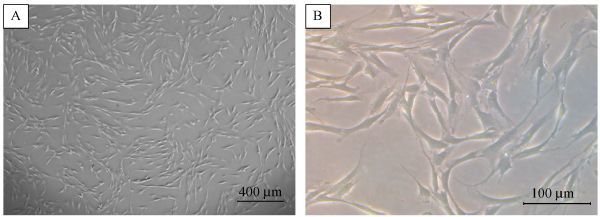
**Morphology of** human periodontal ligament stem cells** (hPDLSCs).**

From PDLSC isolation, several spindle-shaped and fibroblast-like periodontal ligament stem-like cell lines were established (***Fig. 1***). These cells could adhere to plastic and had colony-forming ability. Among these cell lines, the most morphologically appropriate cell line was characterized. Cells were passaged for morphological observation, and the cells over passage 7 showed severe loss of initial morphology. As the passage number increased, the degree of morphological change also increased. Moreover, further passaging of the cells slowed the proliferation rate and increased the cell size.

**Fig.2:**
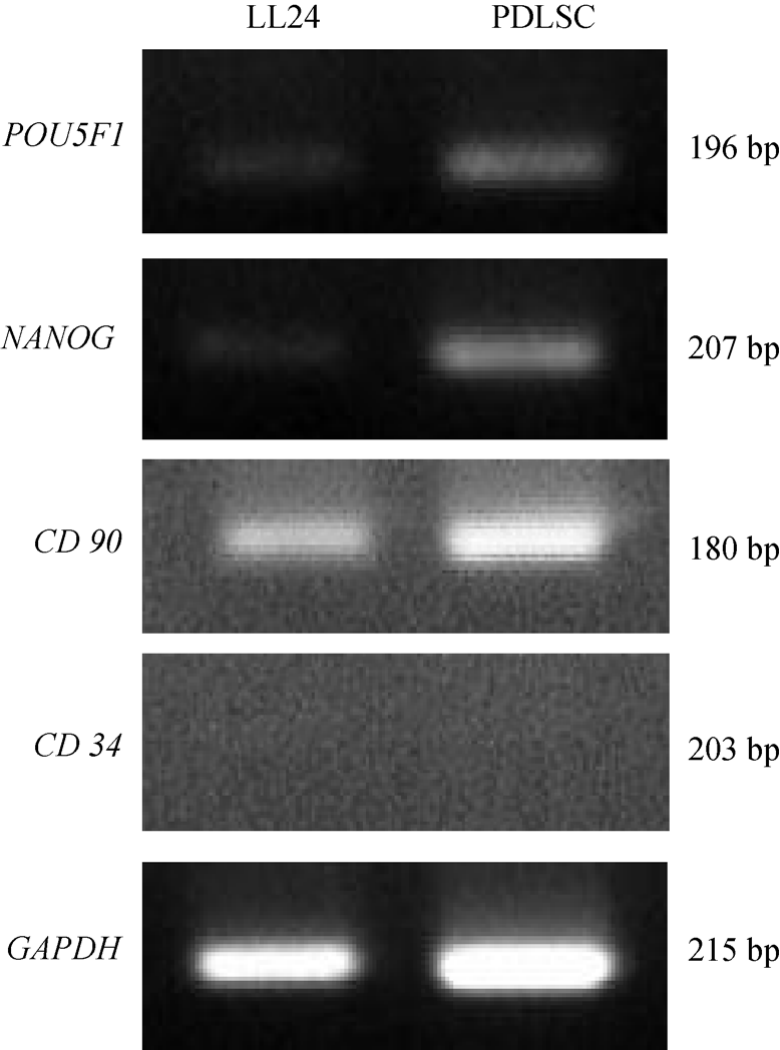
Expression of mesenchymal stem cell markers in periodontal ligament stem cells.

RT-PCR revealed highly expressed markers of undifferentiated, self-renewing, stem cells, such as *POU5F1* (*OCT4*) and *NANOG* in PDLSC (***Fig. 2***). *CD90*, a mesenchymal stem cell marker, also showed high expression. However, *CD73*, a negative marker of mesenchymal stem cells, was not expressed. LL24, a human lung fibroblast, was used as a negative control.

**Fig.3:**
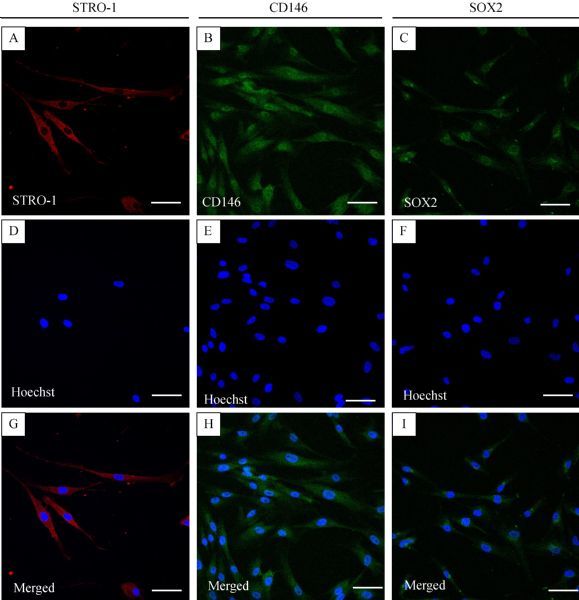
Immunofluorescence analysis of human PDLSCs.

IF was used to evaluate the expression of key mesenchymal stem cell surface markers, including *STRO-1* and *MCAM/CD146*, and the stem cell marker *SOX2* (***Fig. **********3***). Compared with the other markers, *STRO-1* showed very high expression and was expressed throughout the cell (***Fig. 3A–C***). Moreover, *MCAM/CD146* and *SOX2* showed high expression in and near the nucleus rather than in the periphery or margin of the cell (***Fig. 3D–I***).

**Fig.4:**
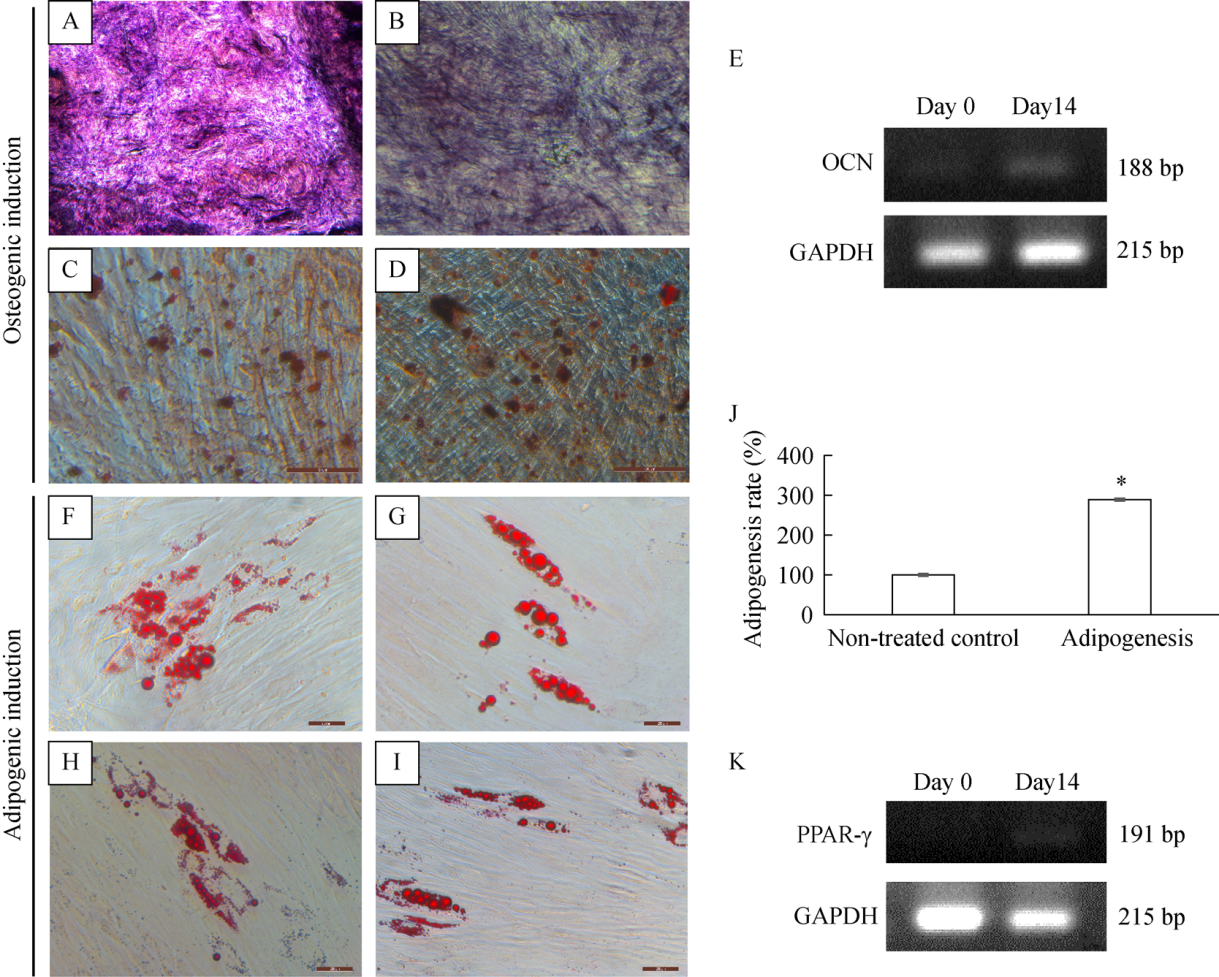
In vitro differentiation of PDLSCs.

Alkaline phosphate staining indicated early osteogenesis as purple-colored cells (***Fig. 4A*** and ***B***). Additionally, mineralized nodules were stained by Alizarin Red S after 14 days of incubation in osteogenic induction medium (***Fig. 4C*** and ***D***). Mineralized nodules were stained a reddish color. The expression of osteocalcin (*OCN*), a key marker of osteogenesis, was slightly increased in the osteogenic group compared with that in the control group (***Fig. 4E***). Lipid droplets were stained by Oil red O after incubation for 21 days in adipogenic induction medium (***Fig. 4F–I***). Stained Oil red O was dissolved with 70% isopropanol, and the OD was evaluated. There was a significant (*P*<0.05) difference between the control group and the 21-day adipogenesis group (***Fig. 4J***). Finally, the expression of peroxisome proliferator-activated receptor γ, an adipogenic marker, was slightly increased in the adipogenesis group compared with that in the control group (***Fig. 4K***). Therefore, these findings confirmed the multiple differentiation potential of PDLSCs.

**Fig.5:**
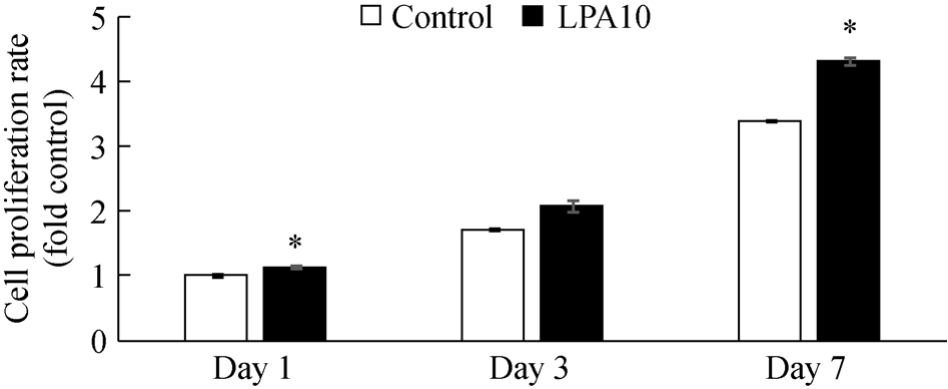
Proliferation rates of PDLSCs treated with 10 μmol/L LPA for 1, 3, and 7 days (P<0.05).

Cells in the group treated with 10 μmol/L LPA showed significantly (*P*<0.05) higher proliferation rates than untreated (control) cells at day 1 (1.12±0.003) and day 7 (4.32±0.005) (***Fig. 5***), and those treated with 10 μmol/L LPA for 3 days showed a tendency toward increased proliferation.

**Fig.6:**
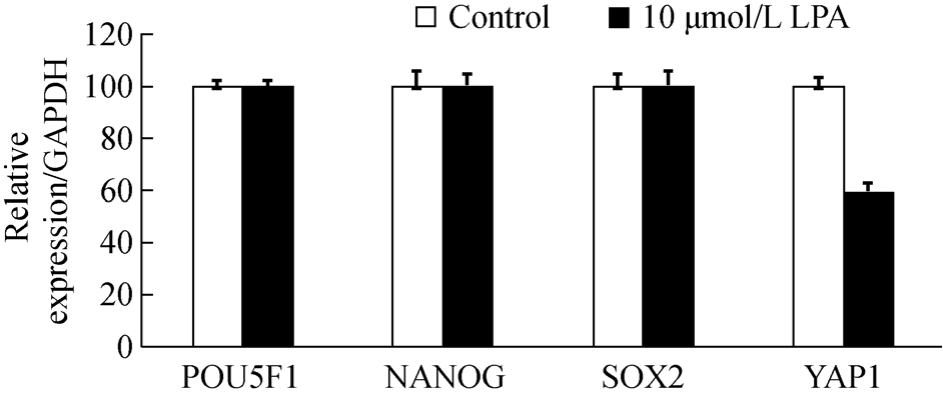
Gene expression changes in PDLSCs after 7 days of culture in the presence of 10 µmol/L LPA, as analyzed by RT-qPCR (P>0.05).

Notably, there were no significant differences in the expression of stemness-related genes such as *POU5F1*, *NANOG*, and *SOX2* after 7 days of culture in the presence of 10 μmol/L LPA (***Fig. 6***). Unexpectedly, *YAP* expression was low in the 10 μmol/L LPA-treated group.******

## Discussion

PDLSCs are human dental stem cells that contain a population of multipotent postnatal stem cells with biological characteristics similar to bone marrow-derived undifferentiated stem cells. PDLSCs can differentiate into periodontal ligament fibroblasts, osteoblasts, and cementoblasts and repair allogenic bone defects without immunological rejection[^31^].

In the present study, we successfully established and characterized PDLSCs isolated from extracted teeth from dental patients. The established cell lines showed expression of *POU5F1* and *NANOG*, the pluripotent markers expressed on PDLSCs[^6^], as well as *CD90*, a mesenchymal stem cell marker. Additionally, the absence of *CD73* expression demonstrated that these cells were not derived from hematopoietic stem cells. At the protein level, although not PDLSC-specific, we also observed expression of the mesenchymal stem cell surface markers *STRO-1* and *CD146*, the valuable indicators of immature mesenchymal stem cells[^11^]. The expression of *SOX2* has been found in PDLSCs as well[^6^]. Taken together, these mRNA and protein analyses demonstrated that the cells were PDLSCs. The efficiency of differentiation potential into multiple lineages *in vitro* was also verified by IF staining and marker gene expression analysis.

Lysophospholipids are bioactive signaling molecules that act through the binding of their specific G-protein-coupled receptors to exert pleiotropic effects on a wide range of cells. The most widely studied signaling lysophospholipids are LPA and sphingosine-1-phosphate (S1P). LPA and S1P can exert developmental, physiological, and pathological effects on the central nervous system and induce biological effects in various types of stem cells[^32^]. Moreover, LPA has been identified as a strong mitogenic factor. Saunders *et al.* reported that LPA increased cell growth and inhibited apoptosis *via* the redox-dependent activation of extracellular signal-regulated serine/threonine kinase-, Akt-, and nuclear factor (NF)-κB-dependent signaling pathways in ovarian cancer cells[^33^].

Furthermore, an association between LPA signaling and reactive oxygen species-mediated signaling has been suggested in other cell types[^34^–^36^]. LPA stimulates the extracellular signal regulated/mitogen-activated protein kinase (ERK/ MAPK) cascade, and is thought to be important for proliferation. LPA increases the proliferation and migration of ASCs *via* Nox4-induced reactive oxygen species (ROS) generation. miR-210 expression is associated with LPA-induced stimulation, and Serpine1 mediates the LPA-induced migration of ASCs. Therefore, phospholipid derivatives, such as LPA and S1P, may be used to stimulate ASCs during stem cell expansion[^37^].

In our previous studies, we showed that treatment with 30 μmol/L LPA during *in vitro* maturation improved the developmental potential of parthenogenic embryos and *in vitro* fertilized embryos in swine. Moreover, LPA enhances the survival of human hematopoietic stem/progenitor cells under ischemic conditions[^17^]. It is also crucial for periodontal wound healing and gingival inflammation[^38^]. LPA enhances the proliferation of human corneal endothelial cells induced by LPA through phosphatidylinositol 3-kinase and Rho-associated protein kinase pathways[^39^].

In a study of embryonic stem cells, Han *et al.* showed that LPA could substitute for YAP to generate transgene-free human naïve pluripotent stem cells[^28^]. Additionally, Cuizhu *et al.* demonstrated that YAP regulated the apoptosis and proliferation of hPDLSCs; specifically, knockdown of the *YAP* gene by small interfering RNA inhibited proliferation, induced apoptosis, and altered the cell cycle of PDLSCs[^40^].

For the application in the dental clinic, expansion of PDLSCs without loss of stemness is essential. In our study, 10 μmol/L LPA promoted the proliferation of PDLSCs. However, there were no significant differences in the expression of stemness-related genes, although *YAP* mRNA tended to be downregulated rather than upregulated. Collectively, our findings demonstrated that LPA enhanced PDLSCs proliferation, but a significant connection between LPA and stemness-related genes in PDLSCs was not identified in this study. Further studies on the mechanisms of LPA-induced PDLSC proliferation and protein expression are needed to clarify the effects of LPA on PDLSCs.

In conclusion, LPA treatmentcan enhance PDLSCs proliferation *via* ROS mediated signaling andpromote cell survival through reversible activation or inhibition of pro-proliferative and regulatory signaling proteins. Thus, LPA treatment of PDLSCs culture may be useful for reprogramming human induced pluripotent stem cells and producing cells for clinical studies of therapeutic regeneration.
